# Laser Remote Sensing of Lake Kinneret by Compact Fluorescence LiDAR

**DOI:** 10.3390/s22197307

**Published:** 2022-09-26

**Authors:** Sergey M. Pershin, Boris G. Katsnelson, Mikhail Ya. Grishin, Vasily N. Lednev, Vladimir A. Zavozin, Ilia Ostrovsky

**Affiliations:** 1 Prokhorov General Physics Institute of the Russian Academy of Sciences, 119991 Moscow, Russia; 2 Department of Marine Geosciences, University of Haifa, Haifa 3498838, Israel; 3 Kinneret Limnological Laboratory, Israel Oceanographic & Limnological Research, Migdal 1495001, Israel

**Keywords:** laser remote sensing, Lake Kinneret, algal blooming, water quality monitoring, chlorophyll-*a* fluorescence

## Abstract

Harmful algal blooms in freshwater reservoirs became a steady phenomenon in recent decades, so instruments for monitoring water quality in real time are of high importance. Modern satellite remote sensing is a powerful technique for mapping large areas but cannot provide depth-resolved data on algal concentrations. As an alternative to satellite techniques, laser remote sensing is a perspective technique for depth-resolved studies of fresh or seawater. Recent progress in lasers and electronics makes it possible to construct compact and lightweight LiDARs (Light Detection and Ranging) that can be installed on small boats or drones. LiDAR sensing is an established technique; however, it is more common in studies of seas rather than freshwater reservoirs. In this study, we present an experimental verification of a compact LiDAR as an instrument for the shipborne depth profiling of chlorophyll concentration across the freshwater Lake Kinneret (Israel). Chlorophyll depth profiles of 3 m with a 1.5 m resolution were measured in situ, under sunlight conditions. A good correlation (R^2^ = 0.89) has been established between LiDAR signals and commercial algae profiler data. A non-monotonic algae depth distribution was observed along the boat route during daytime (Tiberias city–Jordan River mouth–Tiberias city). The impact of high algal concentration on water temperature laser remote sensing has been studied in detail to estimate the LiDAR capability of in situ simultaneous measurements of temperature and chlorophyll concentration.

## 1. Introduction

In recent years, phytoplankton distribution monitoring by various techniques (satellite image processing [1,2,3,4,5,6,7,8,9,10], radar probing [11], laser remote sensing [12,13,14,15,16,17] and contact measurements with sample analysis [18,19,20,21]) has revealed an increased contamination of freshwater [22,23] and seawater areas [24] by blue–green algae. Phytoplankton blooms deplete dissolved oxygen, thus suppressing biodiversity in shallow waters and also poisoning them with biotoxins. This is of particular danger for freshwater reservoirs due to their importance as water supplies for towns and cities. Many cases of toxic algal blooming have been reported worldwide [25,26,27]. For example, Lake Erie has been contaminated by dangerous microcystin toxins due to extensive algal blooming, so NASA carried out airborne hyperspectral imaging of the Great Lakes to reveal the problem scope,- and to detect the most dangerous areas [28]. In Europe, the same problem occurred: a 5–15 cm thick layer of algae was formed during the summer period in coastal zones of Gorky water reservoir on the Volga River in Russia [13]. In 2013, an intense bloom of *Spirogyra* alga was detected in the coastal zone of Lake Baikal [29], which is rather troubling, because Baikal is a freshwater reservoir of international importance as it contains 22% of the world’s fresh surface water [30,31]. Algal blooms have been also monitored in large lakes in Asia by analysis of satellite images [32,33]. It is also known that algal blooms occur frequently near the coasts of California, with chlorophyll concentrations in water exceeding 500 μg per liter [34].

Algal blooms are triggered by increased concentrations of phosphorus and nitrogen minerals carried by natural water currents from agricultural fields [35]. In other words, algal blooms are a good indicator of careless fertilizer utilization by farmers. In turn, water from ponds and reservoirs is used for irrigation, which increases the relevance of water quality monitoring, because toxins may migrate from harmful algae in water to agricultural products as well [36].

Lake Kinneret is a strategic water supply for Israel since it provides fresh water for nearly 2 million people; therefore, water quality monitoring should be performed continuously. In particular, the water level in Lake Kinneret was falling drastically in 2018 [37] due to extensive evaporation (caused mostly by wind) and decreased water income from the Jordan River. Water loss in the lake is accompanied by the growth of organic matter concentration, which in turn may lead to the fast blooming of blue–green algae. Later, the water level in Kinneret recovered due to water intake regulation and favorable weather conditions, but this situation shows that water quality monitoring should be expanded using state-of-the-art remote sensing techniques for better analysis and forecasting.

**Figure 1:**
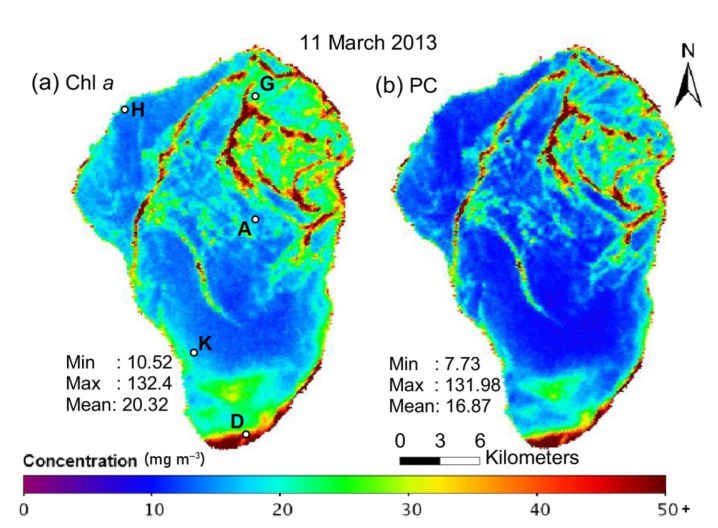
Distribution of (**a**) chlorophyll-*a* (Chl *a*) and (**b**) cyanobacteria-specific pigment phycocyanin (PC) in the surface water layer of Lake Kinneret on 11 March 2013 (calculated using data of the HICO sensor installed at the International Space Station, the image is reproduced from ref. [43]). We have added the location of Stations A, D, G, H and K on panel (**a**).

Kinneret Limnological Laboratory [38] performs regular monitoring of water quality parameters, including the vertical distribution of plankton using direct water sampling and submersible sensors [39]. Typically, such studies are performed once a week at five established points (so-called Stations A, D, G, H and K; see Figure 1a) with a commercial depth profiler to quantify temperature, salinity and chlorophyll-*a* concentration, but some water samples are also collected for more detailed laboratory analysis. These data help to calibrate daytime satellite albedo measurements that represent chlorophyll distribution in the upper water layer of the lake [40,41,42,43].

Figure 1 shows the map of chlorophyll-*a* (Chl *a*) and cyanobacteria-specific pigment phycocyanin (PC) distribution on the lake surface (the image is reproduced from ref. [43]). Such maps are typically constructed from satellite multi- or hyperspectral images [44] in large areas. Still, such images cannot provide important information about the vertical distribution of phytoplankton, which is especially important during cyanobacterial harmful algal blooms [43,45].

Depth-resolved data on phytoplankton concentration are required for the fast detection of toxic cyanobacterial vertical and horizontal distributions and temporal variations. In contrast to satellite sensors, laser radars (also called LiDARs—Light Detection and Ranging) allow for the fast monitoring and mapping of vertical concentrations of phytoplankton from ships, drones or airplanes [46,47]. This technique is suitable for the rapid monitoring of phytoplankton distribution with a high sampling rate and for autonomous operation.

All the LiDARs used for monitoring waterbodies utilize light sources with a wavelength within the water spectral transparency window (450–550 nm). The majority of LiDAR studies describe absorbance measurements [12,47], including papers dealing with satellite-based devices [48,49]. At the same time, there are rather few works utilizing spectral measurements [50,51] due to the complexity of the equipment. Some LiDARs reported in the literature are incapable of daytime measurements [47] due to the use of photomultiplier tube detectors; however, such a disadvantage can be coped with by constructing LiDAR systems based on gated intensified CCD detectors [14].

The aim of this work is to test the possibility of the depth-resolved monitoring of phytoplankton under sunlight conditions, in a freshwater reservoir in real time, using a shipborne compact fluorescence LiDAR instrument.

## 2. Materials and Methods

### 2.1. Location of the Experiment

**Figure 2:**
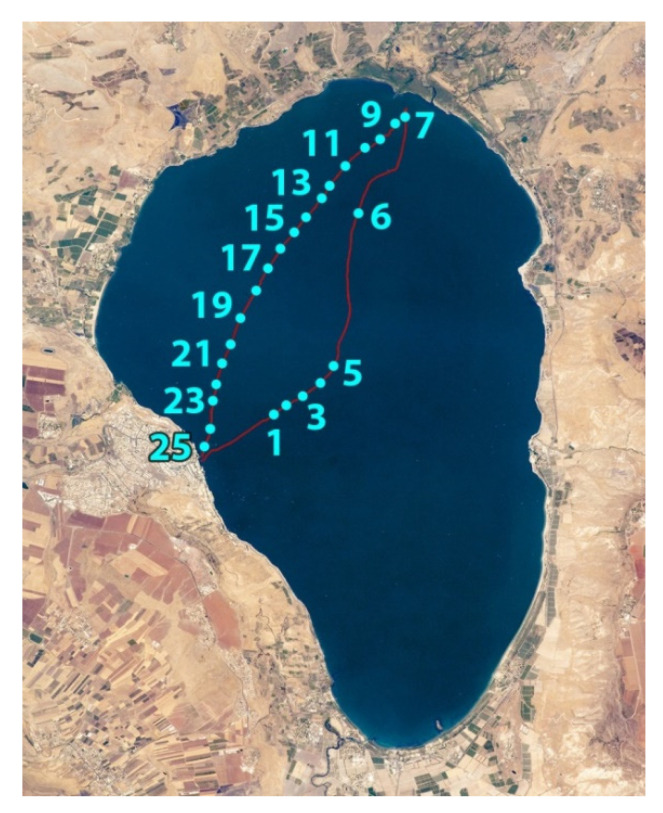
Map of Lake Kinneret with the track of the trimaran boat (red line) and spots where LiDAR sensing was carried out (cyan dots with ordinal numbers).

Experiments were carried out on October 8, 2018, at Lake Kinneret. The compact (25 kg) fluorescence LiDAR (see description below) was installed on a small trimaran boat. Along the boat route from Tiberias city to the Jordan River mouth and back, (Figure 2) water was sensed at 25 spots with the LiDAR to the depth of up to 4.5 m with a 1.5 m depth resolution.

### 2.2. LiDAR

**Figure 3:**
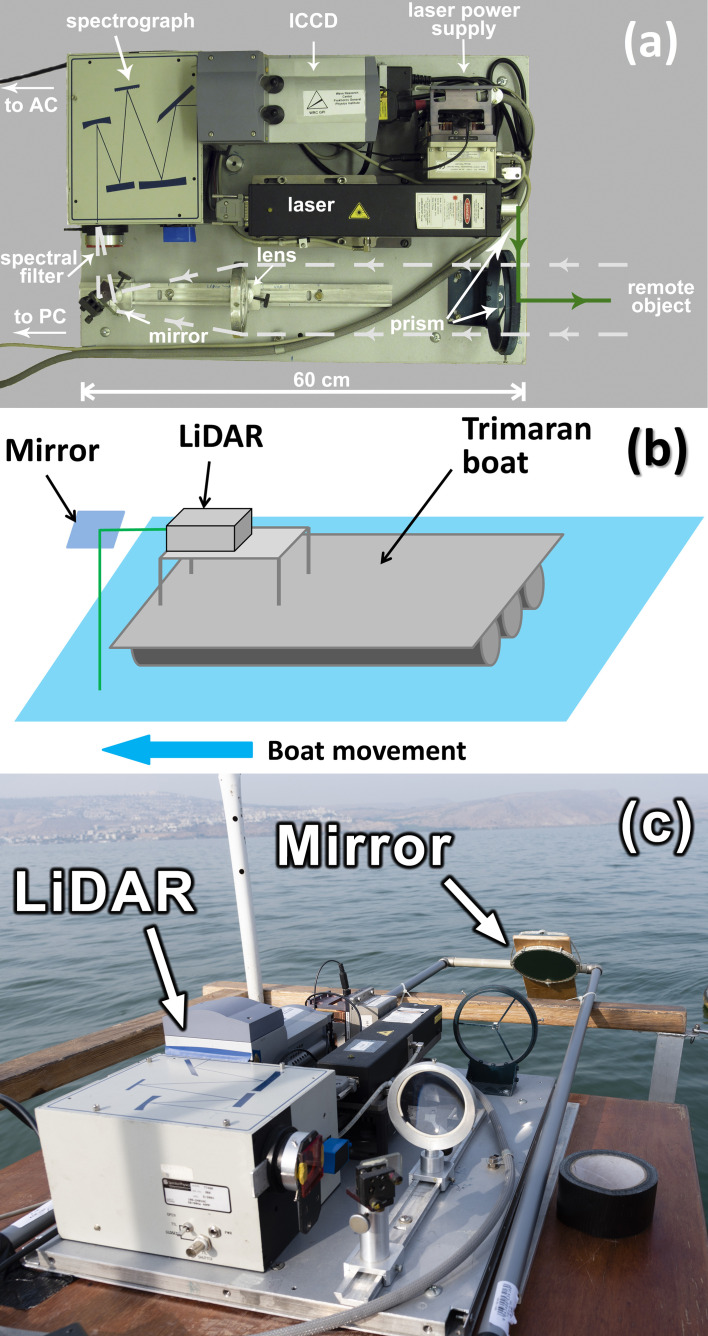
(**a**) Top view of the compact fluorescence LiDAR; (**b**) scheme of the LiDAR sensing; (**c**) photo of the LiDAR installed on the boat.

The fluorescence LiDAR used in our experiments [14] is shown in Figure 3a. It is based on a compact diode-pumped pulsed solid state Nd:YLiF_4_ laser (Laser Compact, Ltd., model LCM-DTL-319QT: 527 nm, 5 ns, 10 Hz, 200 µJ/pulse). Two glass prisms are used to align the probing laser beam and the optical path of the receiving channel. Scattered light from a remote object was collected by a quartz lens (F = 21 cm) to the spectrograph entrance slit. The band-pass glass filters were used to suppress backscattered laser irradiation to prevent detector damage. The detection system consists of a compact spectrograph (Spectra Physics, MS127i) equipped with an ICCD (intensified charge coupled device) camera (Andor iStar). We used a low-resolution diffraction grating (300 lines/mm) and the entrance slit width of 250 µm to increase the signal-to-noise ratio. An aluminum-coated mirror was placed at the boat’s front side to direct the laser beam to the water surface (see the scheme in Figure 3b and the photo in Figure 3c).

The LiDAR’s detection system (ICCD camera) acquired gated spectra with a tunable delay, so scattered/fluorescent photons from different depths could be digitized. The minimal gate step of 3 ns provided ~0.7 m depth resolution, which allowed for measurement of the chlorophyll depth profile.

### 2.3. LiDAR Instrument Calibration

**Figure 4:**
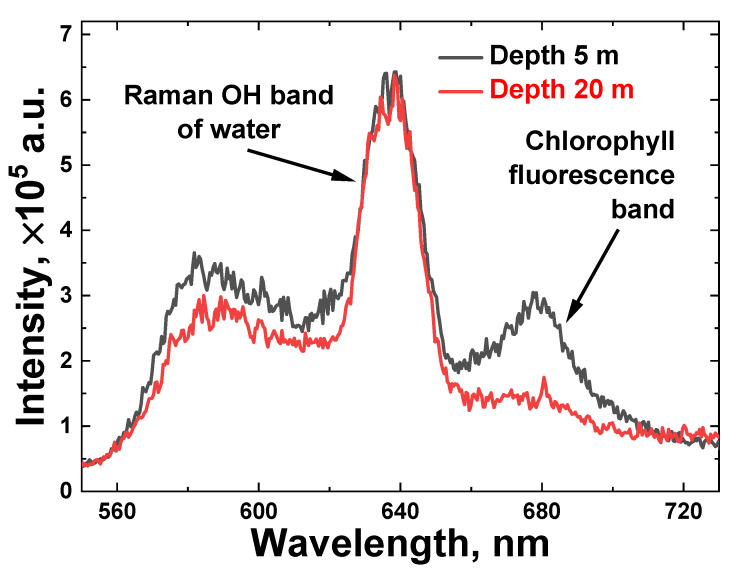
Onboard measured LiDAR raw spectra of water samples taken at station A from 5 and 20 m depths (black and red line, respectively).

First, we carried out LiDAR measurements of water samples taken from different depths near station A (see Figure 1a). Figure 4 illustrates raw LiDAR spectra of two water samples collected from a 5 m and 20 m depth with a Niskin bottle and measured onboard the trimaran. To increase the signal-to-noise ratio, each spectrum was acquired by summing 100 spectra that were detected from single laser pulses. In the spectral acquisition, the laser pulse duration was 10 ns with a 20 Hz repetition rate.

The spectra reflect the combined impact of elastic Mie and Rayleigh scattering of laser pulses, Raman inelastic scattering by water molecules (Raman OH band spectra, 620–650 nm) and chlorophyll-*a* fluorescence (the Chl *a* emission band is 660–700 nm) from algae.

**Figure 5:**
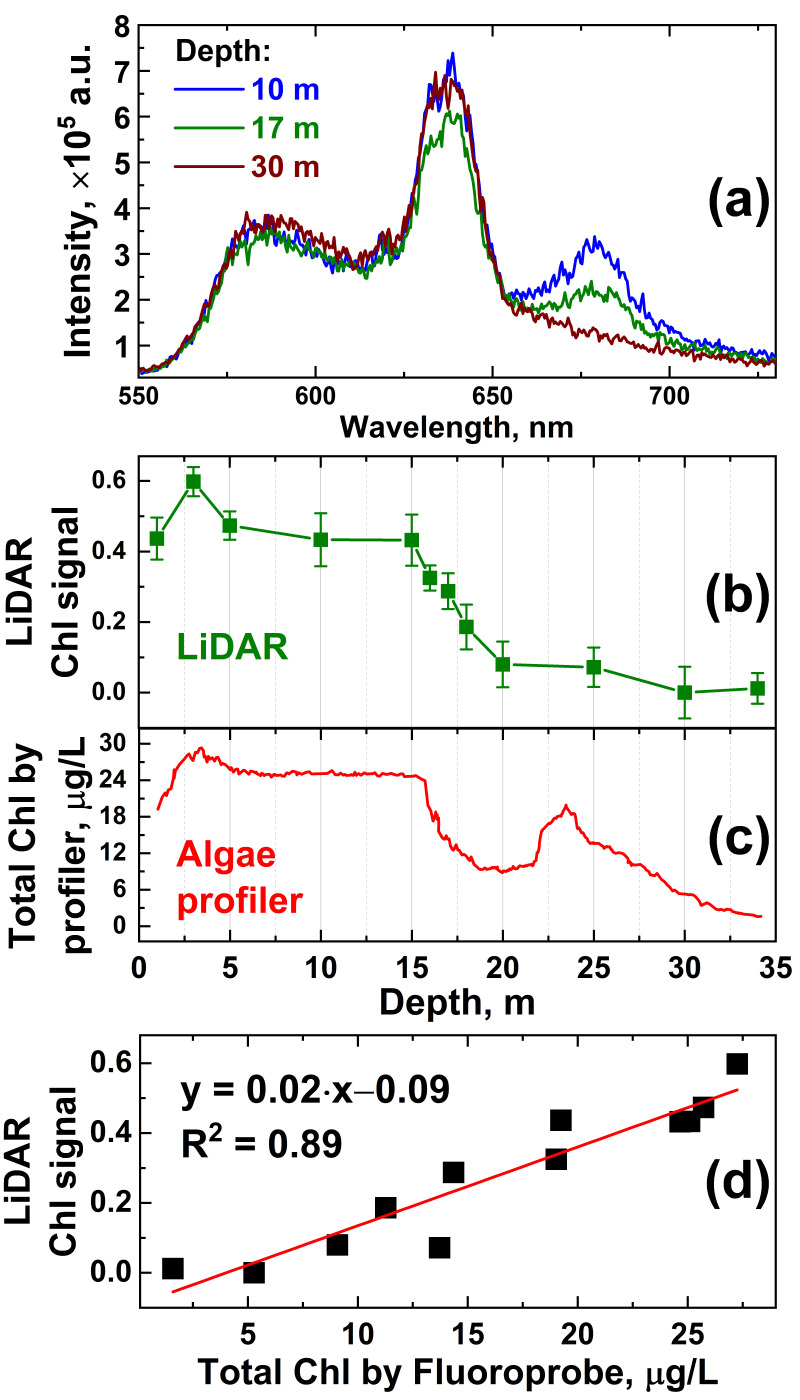
(**a**) LiDAR raw spectra of water samples collected from 10, 17 and 30 m and measured onboard; (**b**) chlorophyll fluorescence signal measured onboard with LiDAR as a function of depth; (**c**) in situ profile of total chlorophyll-*a* concentration measured by the Fluoroprobe profiler; (**d**) scatter plot illustrating the correlation between LiDAR and Fluoroprobe profiler data with a straight line representing the linear fit of the data.

The in situ depth profiling of Chl *a* was carried out with the Fluoroprobe III profiler (bbe Moldaenke, GmbH) for Chl *a* at station A. Concurrently, water samples were collected with the Niskin bottle from different water depths, and then, the onboard measurements were conducted with the fluorescence LiDAR to compare the results. The obtained LiDAR spectra of water collected from 10, 17 and 30 m depths differ noticeably (Figure 5a).

To quantitatively compare in situ LiDAR spectra at different depths, the chlorophyll fluorescence signal should be calculated. Here, we defined LiDAR chlorophyll fluorescence signal $s_{L}$ as follows:

(1) $$s_{L}=\frac{s_{Chl}}{s_{OH}}\;,$$

where $s_{Chl}$ is the background-corrected spectrum integral over the 660–700 nm range, which refers to chlorophyll-*a* fluorescence, and $s_{OH}$ is the background-corrected spectrum integral in the 620–650 nm range corresponding to the Raman OH band. The correction was performed by extracting the linear background. Normalization to the Raman OH band integral allows one to account for the range dependence of the backscattered light intensity for in situ spectra measured with LiDAR, because the Raman scattering intensity represented by the OH band integral is proportional to the amount of H_2_O molecules in the investigated volume [52]. H_2_O is the main constituent in natural water, so the signals of any other admixtures in water should be scaled by the H_2_O molecules’ concentration.

A comparison of the LiDAR chlorophyll signal (Figure 5b) and algae profiler measurements (Figure 5c) revealed a good correlation between the data acquired by the two instruments (R^2^ = 0.89, see Figure 5d) but also confirmed the non-uniform depth distribution of chlorophyll near station A in Lake Kinneret. Consequently, Figure 5 clearly demonstrates the perspectives of LiDAR depth profiling from remotely/automatically driven vehicles when contact measurements are not possible or too time-consuming (12 min vs. 2 min for commercial profiler and LiDAR, respectively). Note that there is a difference between the data of the LiDAR and Fluoroprobe profiler at 20–27 m depths. The total Chl measured with the Fluoroprobe (Figure 5c) combines signals from the Chl *a*-containing algae inhabiting the epilimnion and signals from the bacteriochlorophyll *e*-containing phototrophic bacteria dominating the metalimnion [53,54], i.e., in our case, the water between the 22 m and 27 m isobaths. However, phototrophic bacteria fluoresce in the ~720–850 nm region [55], which is almost beyond the spectral sensitivity window of our LiDAR (500–750 nm), so the contribution of phototrophic bacteria at 20–27 m depths to the LiDAR signal was minimal (Figure 5b).

**Figure 6:**
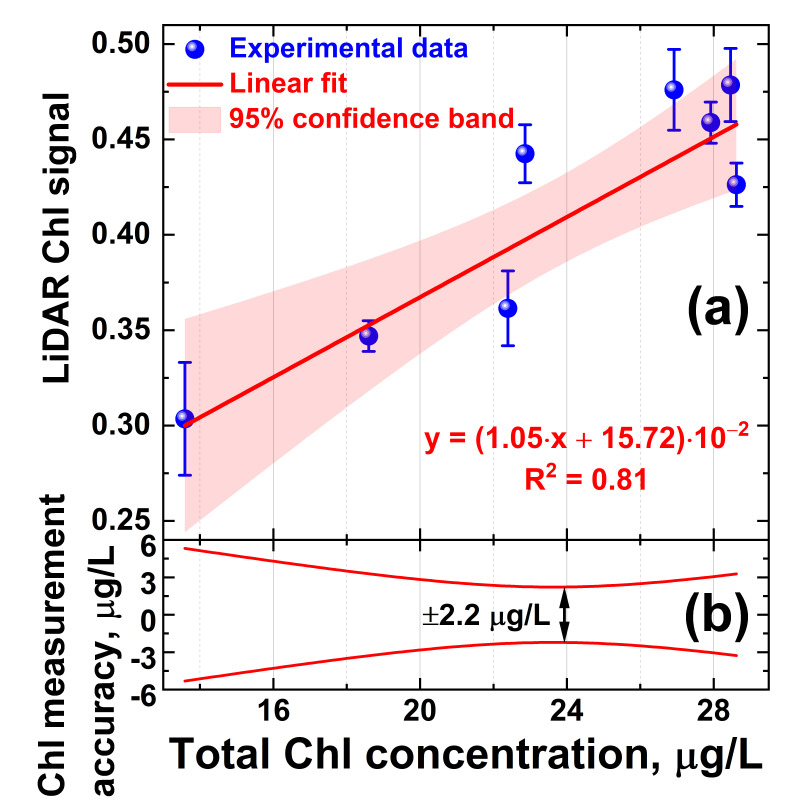
(**a**) LiDAR calibration curve for chlorophyll concentration measurements; (**b**) accuracy of remote chlorophyll concentration measurements by LiDAR defined as abscissa interval between the upper and lower confidence band curves at the selected ordinate value. Vertical bars in the panel (**a**) show standard deviation of the parallel measurements.

Owing to the correlation of the algae profiler and LiDAR measurement results, the LiDAR was calibrated for quantitative Chl *a* measurements. Typically, LiDAR data are calibrated by water samples taken at LiDAR measurement points and later analyzed in the laboratory using certified techniques [56]. However, the Chl *a* concentration can be altered during phytoplankton transportation to the laboratory over a few hours [57], so systematic errors may be introduced into the LiDAR calibration. Here, we carried out in situ LiDAR instrument calibration on the total chlorophyll concentration. A series of 8 water samples was taken with the Niskin bottle from 1, 3, 5, 10, 14, 16, 17, and 18 m depths at Station A. These samples were immediately measured onboard the trimaran with the LiDAR. Reference values of the total chlorophyll-*a* concentration were acquired in situ during the Fluoroprobe vertical profiling performed concurrently with water sampling. Given the above-mentioned difference between the LiDAR and Fluoroprobe data at 20–27 m depths (Figure 5b,c, respectively), for proper LiDAR calibration, the data collected below the 20 m isobath were not used in the calibration to avoid the impact of the bacteriochlorophyll *e* on the total Chl readings by the Fluoroprobe profiler. Five parallel LiDAR measurements were carried out for each sample to calculate standard deviations. The dependence of the LiDAR chlorophyll-*a* signal versus Fluoroprobe measurements is presented in Figure 6. The LiDAR calibration curve was obtained by linear fitting, and 95% confidence band intervals were computed to evaluate measurement errors. Here, we define the accuracy of chlorophyll concentration measurements by LiDAR as the corresponding abscissa interval between the upper and lower confidence band curves at the selected ordinate value. The error of chlorophyll-*a* concentration measurements by the algae profiler was ±0.01 μg/L, according to the manufacturer’s specification [58]. The results suggest that chlorophyll concentration measurements by LiDAR were possible with accuracy of ±2.2 μg/L (Figure 6b).

The LiDAR instrument calibration is based on an assumption that the lateral and horizontal algal parameters are similar in quality, but different only in quantity. This assumption can be accepted for our detection method because different algal types may differ by the shape of the fluorescence band, which has no effect on the fluorescence signal as we define it (band integral). Additionally, the LiDAR has a broadband detector, so changes in the fluorescence band position are not crucial as well.

## 3. Results and Discussion

### 3.1. LiDAR Monitoring of Chlorophyll Concentration

**Figure 7:**
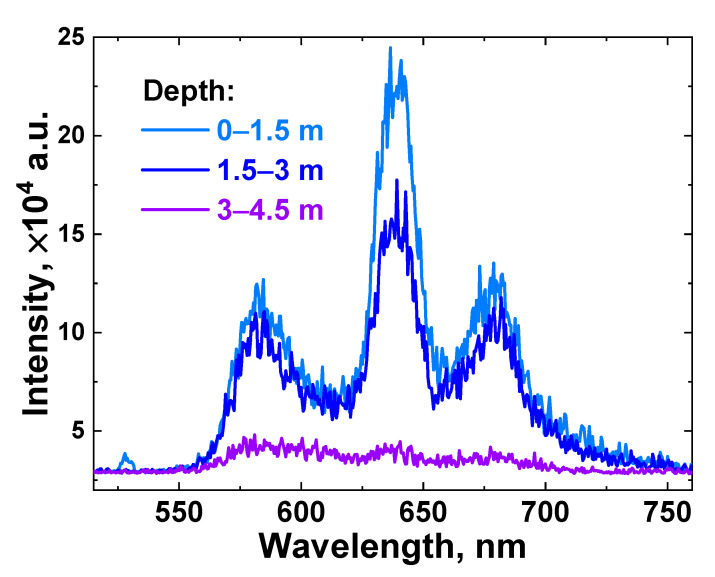
LiDAR raw spectra acquired in situ at different depths (light blue line—0–1.5 m; blue line—1.5–3 m; and violet line—3–4.5 m) in the vicinity of measurement spot №15 (see Figure 2).

After calibrating the LiDAR instrument, we performed in situ laser remote sensing of the surface water layer in Lake Kinneret. Due to limited time and resources in field experiments, this study considered limited data necessary for the experimental verification of the LiDAR freshwater reservoir monitoring technique. Examples of raw LiDAR spectra detected in situ from different depths are presented in Figure 7.

The acquired spectrum intensity fell as the depth increased, and for a 3–4.5 m depth layer, the signal bands had very low intensity due to a strong scattering of the laser radiation in turbid water; hence, no meaningful information could be obtained from it, and further, this layer will be excluded from consideration.

**Figure 8:**
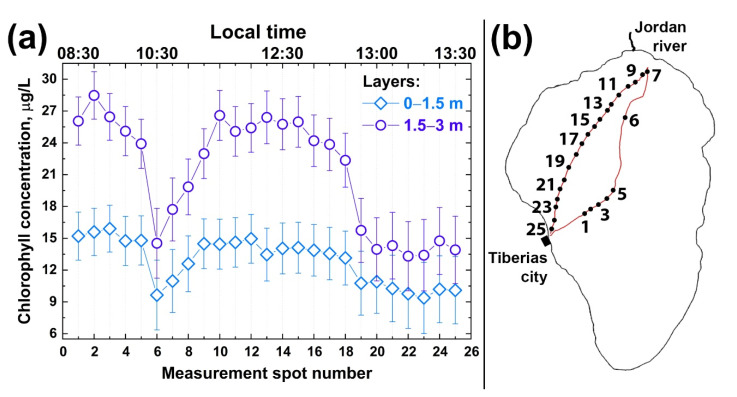
(**a**) Chlorophyll-*a* concentration profiles in Lake Kinneret at two depth intervals: 0–1.5 m (rhombuses) and 1.5–3 m (circles), measured by fluorescence LiDAR on 8 October 2018 in different spots along the trimaran route shown on the panel (**b**). Measurement spot numbers on the panel (**a**) correspond to ones on the panel (**b**).

First, we studied the depth profiles of chlorophyll concentration at different points. Figure 8a illustrates the results of chlorophyll concentration depth profiling along the trimaran route from Tiberias city to the Jordan River mouth and back (see the route in Figure 8b). According to Figure 8a, the maximum chlorophyll concentration was observed at a depth of 1.5–3 m. The chlorophyll concentration was significantly lower in the upper water layer, which may be explained by phytoplankton diurnal vertical migration [59,60]. The comparison of different depth horizons indicates that chlorophyll concentration in the first and second layers (0–1.5 m and 1.5–3 m depth) changed symbatically along the sensing route. Several features can be outlined: (i)—chlorophyll concentration in the first layer was twice as low as in the second one, so the algae distribution over Lake Kinneret derived from satellite images may not be correct; (ii)—chlorophyll concentrations near Tiberias city and near the Jordan River mouth were twice as high as in the middle of the Lake; (iii)—chlorophyll concentrations in the first and second water layers varied symbatically.

It can be seen from Figure 8a that the chlorophyll concentration was rather high at depths of up to 3 m near the Tiberias beach (points 1–4 in Figure 8b). Near the Ecoraft monitoring station (point 5 in Figure 8b) [39], the fluorescence signal was lower (point 6 in Figure 8b). When moving to the Jordan River mouth, the chlorophyll concentration began to increase in 0–1.5 m and 1.5–3 m layers and, like near Tiberias city, differed two-fold between the layers (~14 and ~26 µg/L, respectively). Near the Jordan River mouth, the decreased chlorophyll concentration was observed in 1.5–3 m and 0–1.5 m layers. This near-shore area with a decreased chlorophyll-*a* concentration was about 800 m wide. When the trimaran moved away from the Jordan River mouth towards Tiberias, the chlorophyll concentration fell almost two-fold in both layers (points 15 to 24).

Our experiments on the LiDAR non-contact remote sensing of algae chlorophyll performed over several hours (08:30–13:30) clearly indicate the non-uniformity of algal distribution in Lake Kinneret. Quenching could be an apparent reason for lower concentration of Chl *a* near the water surface measured with both instruments (the fluorescence LiDAR and Fluoroprobe). Therefore, remote quantification of Chl *a* in the deeper (1.5–3 m) layer with the fluorescence LiDAR could provide an unbiased assessment of the Chl *a* concentration in the upper well-mixed epilimnion.

### 3.2. Water Temperature Sensing by LiDAR under High Algae Concentration

The remarkable feature of the LiDAR instrument is its capability to measure water temperature, including depth profiling by quantifying Raman OH band profile distortion [61,62]. Water temperature is one of the important parameters in ecological surveys of water bodies. There are numerous techniques and instruments for the remote sensing of water temperature including LiDARs, and descriptions of these techniques can be found elsewhere [62,63]. However, in the majority of works, water temperature is studied in seas where the algal concentration is low compared to that of freshwater reservoirs, which makes LiDAR measurements of seawater temperature rather easy. Typically, the non-bloom algal concentration in a marine environment is <1000 cells/mL [64], while in freshwater reservoirs, it may exceed 30 000 cells/mL [65]. Here, we conducted a systematical study of high algal concentration influence on LiDAR water temperature measurement accuracy. Recently, we have demonstrated that the laser remote sensing of temperature can reach 0.15 °C accuracy for distilled water [62]. The suggested “centroid” technique is very sensitive to small distortions of the Raman OH band envelope; however, we have not studied the impact of the Chl *a* fluorescence peak on the accuracy of temperature measurements.

**Figure 9:**
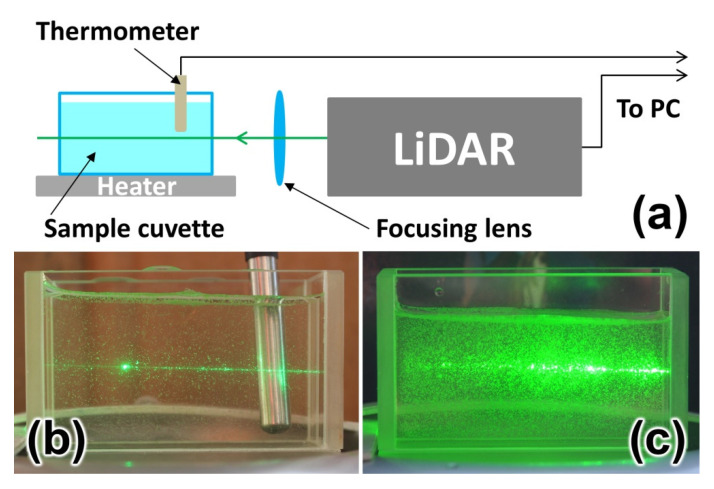
Experimental scheme of water temperature measurements by LiDAR (**a**); photograph of the cuvette with a sample having 420 cells/mL algae concentration (**b**); photograph of the cuvette with a sample having 10,800 cells/mL algae concentration (**c**).

In order to investigate the impact of high algal concentration on the accuracy of water temperature measurements by LiDAR, we carried out a systematic study. Natural water samples with high algal concentrations were placed in a glass cuvette installed 0.5 m from the LiDAR (Figure 9). The cuvette was cooled for 2 minutes down to 7 °C in a small refrigerator and then placed on a heater plate. LiDAR measurements were performed during cuvette heating until a sample temperature of 27 °C was achieved (controlled by a DS18B20 digital thermometer with ±0.1 °C accuracy). The heating took 10 minutes, so phytoplankton should not have changed their properties during the measurements.

**Figure 10:**
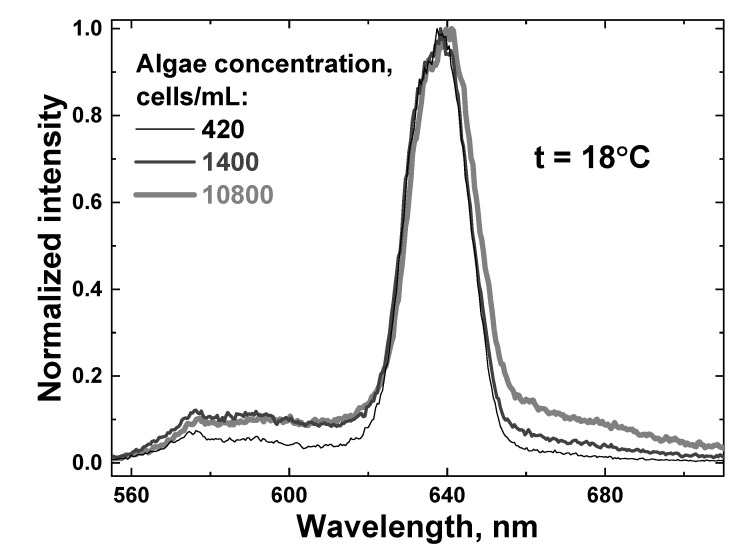
Raw LiDAR spectra of water samples with different algae concentrations: 420 cells/mL (thin black line), 1400 cells/mL (dark gray line) and 10,800 cells/mL (thick light gray line). All the spectra were recorded at the sample temperature of 18 °C.

Afterwards, a new portion of natural water was taken, and the algal concentration was decreased by adding an appropriate amount of distilled water. Then, the sample was cooled down to 7 °C, and the heating experiments were repeated. A series of water samples with different algal concentrations were measured (420, 840, 1400, 4300 and 10,800 cells/mL, measured by the YSI 6600 v2 profiler, YSI Inc., Yellow Springs, OH, USA), and examples of LiDAR spectra are shown in Figure 10.

**Figure 11:**
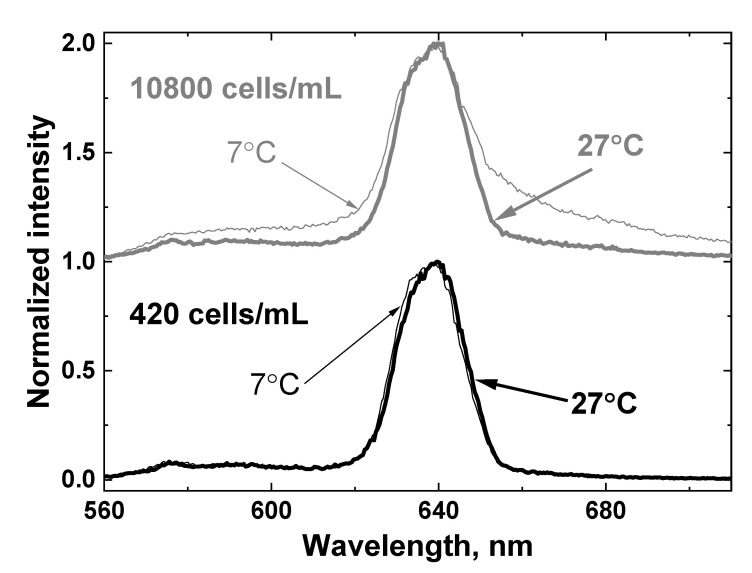
LiDAR spectra of two samples with minimal and maximal algae concentration: 420 cells/mL (lower panel) and 10800 cells/mL (upper panel) at low and high temperature (7 and 27 °C). The spectra are shifted vertically for better view.

Figure 11 shows the LiDAR spectra of two samples with minimal and maximal algal concentrations (420 and 10,800 cells/mL, correspondingly) at low and high temperatures (7 and 27 °C). It can be seen that both the temperature and presence of algae distort the OH band contour.

**Figure 12:**
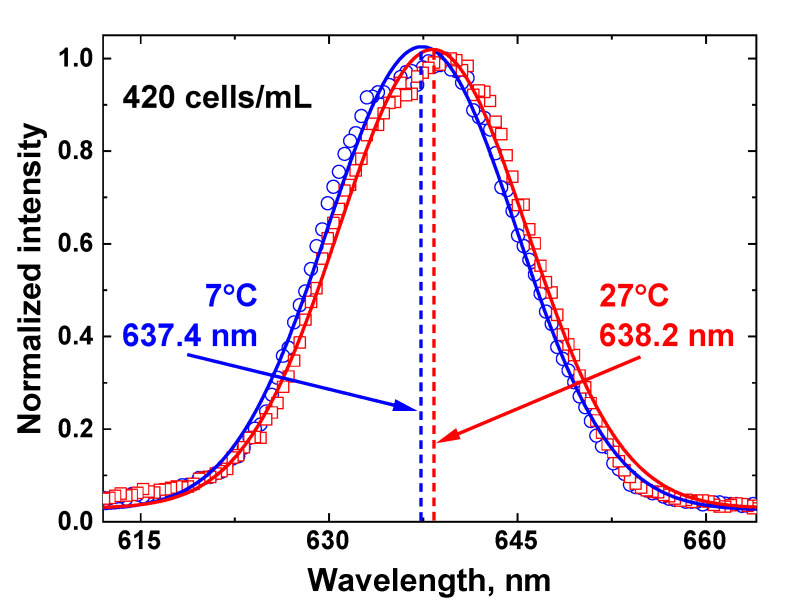
An illustration of the centroid technique for remote water temperature measurements of the water sample with algae concentration 420 cells/ml. The Raman OH band recorded by the LiDAR (blue circles for sample temperature 7 °C and red squares for 27 °C) is fitted with a Gaussian curve (solid lines), and the fitting curve center (represented by dashed lines) is a temperature-dependent metric of the centroid technique.

The centroid technique temperature measurements are based on the non-linear fitting of the Raman OH band by a Gaussian curve, so any changes in the OH band envelope will influence the fit results. An example of Raman OH band fitting by the centroid technique for the 420 cell/mL concentration at two different temperatures is presented in Figure 12.

**Figure 13:**
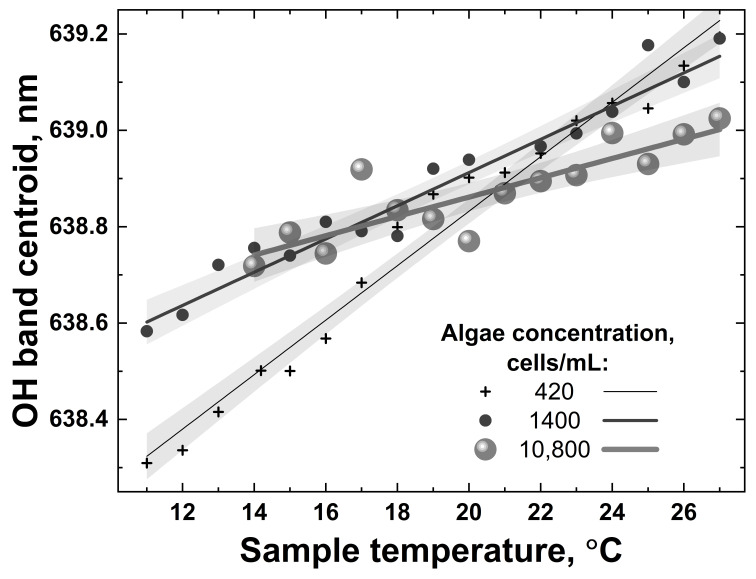
Examples of temperature dependencies of the OH band centroid for water samples with different algae concentrations (420, 1400 and 10,800 cells/mL) when heated from 10 °C to 27 °C. Symbols represent OH band centroids calculated from the experimental spectra; solid lines and shaded regions are corresponding linear fits and their confidence bands.

The LiDAR OH band spectra recorded in the experiment for different sample temperatures (for example, 7 and 27 °C in Figure 12) were fitted with Gaussian curves, and after that, the temperature dependence of the fitting curve center was plotted. The detected spectra were further processed using the centroid technique, and the results are presented in Figure 13.

**Figure 14:**
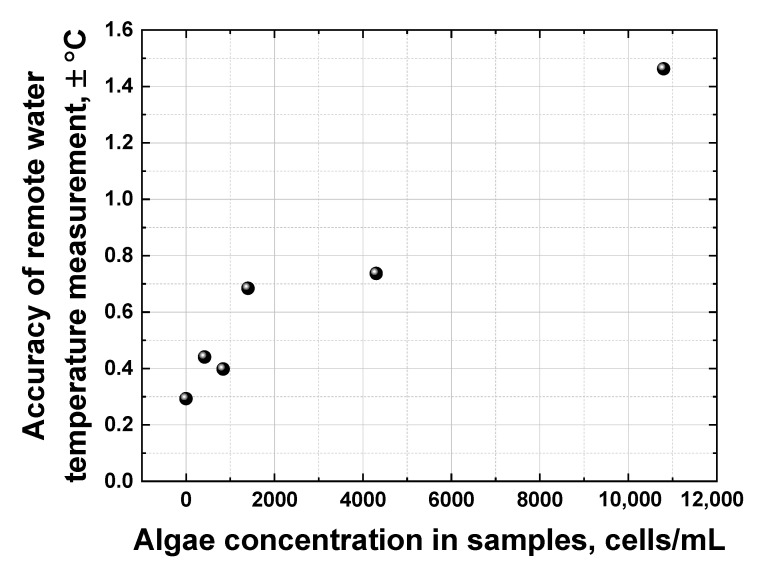
Dependence of the remote water temperature measurement accuracy on algae concentration in samples.

All the dependencies in Figure 13 are close to linear; however, they have different slopes and different scatters. The accuracy of remote LiDAR water temperature measurements was defined as the corresponding abscissa interval between the upper and lower confidence band curves at the selected ordinate value (confidence bands were plotted with a 0.95 probability level). Figure 14 summarizes the results of water temperature measurement accuracy calculations for samples with different algal concentrations (accuracy for distilled water temperature measurement has also been added for comparison, the point at 0 cells/mL in Figure 14).

Figure 14 clearly indicates that the accuracy of remote water temperature measurements changes almost linearly as a function of algal concentration. However, at ~11,000 cells/mL, the chlorophyll fluorescence spectral band starts to overlap with the Raman OH band of water, leading to a drop in temperature measurement accuracy down to approx. ±1.5 °C. A further increase in algal concentration in water makes it impossible to distinguish the OH band correctly and to calculate its centroid to measure the water temperature.

Thus, laser remote sensing is a good technique for expressing the assessment of water quality including depth-resolved measurements. The LiDAR instrument can be further improved in terms of sensing depth and compactness by using a set of sensitive single-point photodetectors (e.g., single-photon avalanche diodes) with band-pass optical filters suited for specific spectral bands of fluorescence and elastic scattering. Such an improvement would allow lightweight LiDAR instruments to be made, which opens a possibility of algal concentration monitoring using unmanned vehicles (e.g., boats or multicopters) [66].

## 4. Conclusions

For the first time, to the best of our knowledge, the laser remote sensing of Lake Kinneret (Israel) has been carried out utilizing a compact shipborne fluorescence LiDAR based on a pulsed Nd: YLF laser (5 ns, 527 nm, 200 μJ/pulse, 10 Hz) and a spectrometer equipped with a gated CCD camera, providing 0.7 m depth resolution. The LiDAR has been experimentally verified as an instrument for depth-resolved water quality monitoring in terms of algal concentration and water temperature. Chlorophyll fluorescence spectra have been quantified in situ of up to 3 m in depth along the ship route under sunlight conditions, and a good correlation has been established (R^2^ = 0.89) between the LiDAR chlorophyll signal and the commercial algae profiler data. After the LiDAR calibration, the maximum accuracy of the remote LiDAR chlorophyll concentration measurements has been estimated as ±2.2 μg/L. A non-monotonic algal depth distribution has been detected along the ship route during daytime, indicating the importance of depth-resolved measurements. The impact of a high algal concentration on the accuracy of water temperature measurements has been systematically studied: the accuracy of water temperature measurements drops from ±0.3 °C to ±1.5 °C as algal concentration increases from 0 to 11,000 cells/mL.

## Data Availability

Not applicable.
